# Establishing a core outcome measure for cancer in trials in kidney transplantation: a standardized outcomes in nephrology-kidney transplantation consensus workshop report

**DOI:** 10.3389/ti.2026.16181

**Published:** 2026-06-08

**Authors:** Ellen Dobrijevic, Anita van Zwieten, Allison Jaure, Armando Teixeira-Pinto, Sergio A. Acuna, Curie Ahn, Eric Au, Christopher Blosser, Jonathan C. Craig, Dale Coghlan, Bianca Davidson, Beatriz Dominguez-Gil Gonzalez, Anna Francis, Chandana Guha, Martin Howell, Anastasia Hughes, Kenar D. Jhaveri, Kenneth A. Newell, Jolanta Malyszko, Alejandra Mena-Gutierrez, Naoka Murakami, Colm O'Reilly, Javier Recabarren-Silva, Dharshana Sabanayagam, Nicole Scholes-Robertson, Amanda Sluiter, Helio Tedesco Silva Junior, Andrea K. Viecelli, Germaine Wong

**Affiliations:** 1 School of Public Health, The University of Sydney, Darlington, NSW, Australia; 2 Children’s Hospital at Westmead Centre for Kidney Research, Westmead, NSW, Australia; 3 Department of Surgery, Division of Transplantation, The University of Alabama at Birmingham, Birmingham, AL, United States; 4 Seoul National University, Gwanak-gu, Republic of Korea; 5 Melbourne School of Population and Global Health, The University of Melbourne, Parkville, VIC, Australia; 6 Renal Department, The Alfred, Melbourne, VIC, Australia; 7 Department of Medicine, University of Washington, Seattle, WA, United States; 8 College of Medicine and Public Health, Flinders University, Adelaide, SA, Australia; 9 Division of Nephrology and Hypertension, Groote Schuur Hospital, Observatory, South Africa; 10 Organizacion Nacional de Trasplantes, Madrid, Spain; 11 Department of Nephrology, Queensland Children’s Hospital, South Brisbane, QLD, Australia; 12 Northwell Health, New Hyde Park, NY, United States; 13 Donald and Barbara Zucker School of Medicine at Hofstra/Northwell, Hempstead, NY, United States; 14 Emory University School of Medicine, Atlanta, GA, United States; 15 Department of Nephrology, Dialysis and Internal Medicine, Warszawski Uniwersytet Medyczny, Warsaw, Poland; 16 Wake Forest University School of Medicine, Winston-Salem, NC, United States; 17 Department of Medicine, Washington University in St Louis, St. Louis, MO, United States; 18 Nephrology Division, Universidade Federal de Sao Paulo, São Paulo, Brazil; 19 Hospital do Rim, Fundação Oswaldo Ramos, São Paulo, Brazil; 20 Australasian Kidney Trials Network, The University of Queensland, QLD, Brisbane, Australia; 21 Department of Nephrology, UniversitatsSpital Zurich, Zürich, Switzerland; 22 Centre for Transplant and Renal Research, Westmead Hospital, Westmead, Australia

**Keywords:** cancer, chronic kidney disease, core outcome measure, kidney transplantation, patient-centered outcomes

## Abstract

Cancer is a critically important outcome for kidney transplant recipients that inconsistently and infrequently reported in trials. We convened a consensus workshop to establish a core outcome measure for cancer for trials in kidney transplant recipients. Workshop attendees included 21 transplant recipients, 2 caregivers and 46 health professionals from 12 countries. Transcripts were analyzed thematically. Three themes were identified. “*Fear of cancer occurrence due to immunosuppression*” reflected the psychological burden and uncertainties when balancing suppression with long-term cancer risk. “*Capturing the details of type, stage and recurrence*”, encompassed recognizing the differential consequences of cancer types, delineating the stage of cancer to convey severity, and distinguishing recurrent from *de novo* cancer. “*Recognizing the challenges of capturing cancer events*” included under-reporting and incomplete documentation of longer-term outcomes, variability in cancer screening practices, and absence of coordinated trial networks to support harmonization and aggregation of cancer outcomes across studies. Participants agreed that occurrence, with stratification by cancer type, stage, and recurrence, where possible, would be a practical and meaningful core outcome measure for cancer. Consistent reporting of cancer using a standardized core outcome measure may help to improve the consistency and relevance of trial findings and assist in shared decision-making in kidney transplantation.

## Introduction

Cancer is a leading cause of mortality and morbidity in kidney transplant recipients, who have a 2-3-fold increased risk of developing cancer and cancer-related death compared to the age- and sex-matched general population [1, 2]. Such elevated risk is primarily attributed to the effects of immunosuppression, which impair immune surveillance, disrupts normal tumor elimination mechanisms and thus increases susceptibility to virus and non-virus-related cancers [3–5]. With advancements in kidney transplantation leading to improved long-term graft survival, cancer is an increasing problem for patients and the health system, representing a significant barrier to long-term survival in kidney transplant recipients.

The Standardized Outcomes in Nephrology-Transplantation (SONG-Tx) international consensus process involving patients, caregivers and health professionals has identified cancer as a critically important core outcome to be reported in all trials in kidney transplant recipients, along with graft health, cardiovascular disease, infection, life participation and mortality [6, 7]. Despite this, cancer is infrequently reported in trials, with highly variable definitions, terminology, metrics and time points used. Of 819 trials in kidney transplantation published between 2000 and 2021, only 84 (10%) reported a cancer outcome, with 72 different cancer outcome measures used [8]. These limitations in how cancer is reported in trials restrict the ability to compare or aggregate data between trials in kidney transplantation, and thus impair decision-making about interventions to address cancer in this population.

A core outcome measure can help to ensure consistent reporting of cancer in trials to improve the comparability, relevance and use of evidence [9, 10]. To inform this work, an international SONG-Tx survey was conducted with 403 patients, caregivers and health professionals from 55 countries to prioritize cancer outcomes identified through a systematic review and consultation with the expert working group [11]. Stakeholders consistently identified the occurrence of cancer, death from cancer and impact of cancer on graft function as the most critical cancer domains for trials in kidney transplant recipients. As mortality and graft health are also core outcomes identified by SONG-Tx, cancer occurrence is the sole remaining, highest priority outcome not captured in the core outcome set [7]. To advance this work, an international workshop was convened to establish consensus on defining and implementing cancer occurrence as a core outcome measure for cancer in trials in kidney transplantation. We aimed to summarize the perspectives of patients, caregivers, and health professionals shared during the workshop and present the key recommendations for the development and implementation of a core cancer outcome and relevant metric in trials of kidney transplant recipients.

## SONG-Tx Cancer workshop

### Context and scope

The one-hour SONG-Tx Cancer Consensus Workshop was held using Zoom videoconference on May 21, 2024. The workshop was informed by a systematic review of cancer outcomes reported in trials in kidney transplant recipients [8], and an international survey in which patient, caregivers and health professionals prioritized cancer outcomes for trials in kidney transplantation [11]. The aim was to bring together insights from patients, caregivers and health professionals and discuss the identification and implementation of a core outcome measure for cancer in clinical trials. The study was approved by the human research ethics committee of University of Sydney (2015-228).

### Workshop attendees and contributors

Sixty-nine stakeholders attended the workshop, including 21 transplant recipients, 2 caregivers and 46 health professionals (adult and pediatric nephrologists, other physicians, surgeons, nurses, and researchers). The attendees were from 12 countries, with 31 from Australia, 24 from the United States of America, 3 from Canada, 2 from each of Hong Kong and the United Kingdom, and one participant from each of France, Indonesia, Italy, Malaysia, Mexico, Switzerland and Turkey. We sent targeted invitations to health professionals with clinical expertise in cancer in kidney transplantation (identified by the SONG-Tx expert working group and through previous SONG activities). Patients and caregivers were invited by members of the SONG-Tx Cancer expert working group. 133 individuals were invited to participate. The full list of SONG-Tx Cancer workshop attendees and contributors is provided in the Supplementary Table S1.

### Workshop program and materials

Background materials and the workshop program were sent to all facilitators and co-facilitators 1 week before the workshop. The question guide for the workshop facilitators and definitions for cancer outcomes were included in the Supplementary Tables S2, S3. The workshop commenced with a brief presentation introducing the SONG Initiative and core outcomes, the results from the systematic review and survey on cancer in kidney transplantation, and the proposed outcome measure with justification [8, 11]. The presentation was followed by facilitated discussions in pre-allocated breakout groups, selected to maximize diverse discussions and broad knowledge exchange among different stakeholders. There were seven breakout groups, each comprising 9–11 members, with at least one SONG-Tx Cancer expert working group member and three patients or caregivers in each group. Each group was led by a facilitator (G.W., D.S., N.S.R., A.V., E.D., J.C. and A.vZ.) who prompted the discussion using a question guide developed by the SONG-Tx Cancer expert working group and investigators (Supplementary Table S2). The questions covered: should the occurrence of cancer be the core outcome measure, are there any other important aspects that should be included in a core outcome measure, and are there any suggestions to encourage and support the implementation of this measure in trials? The breakout groups reconvened in the final plenary session, where a volunteer from each group provided a brief summary of their discussion. The Chair (G.W.) moderated this session and provided a summary of the key points from all groups and closing remarks.

All plenary and breakout discussions were recorded and transcribed verbatim. The transcripts were then imported into HyperRESEARCH (ResearchWare Inc. United States; Version 4.5.7) software for coding and thematic analysis of the data. First author (E.D.) read and coded each transcript line by line to identify themes and subthemes related to determining the core outcome measure for cancer. The themes were reviewed by A.v.Z, G.W. and A.J. A draft workshop report was distributed to all participants and non-attending contributors and feedback was invited within a 2-week timeframe. Additional comments were integrated into the final report to ensure that the findings reflected participants’ perspectives and opinions. The recommendations for implementation table were generated from the content of the workshop as well as from discussion and review by the expert working group.

### Synthesis of workshop discussion

**FIGURE 1:**
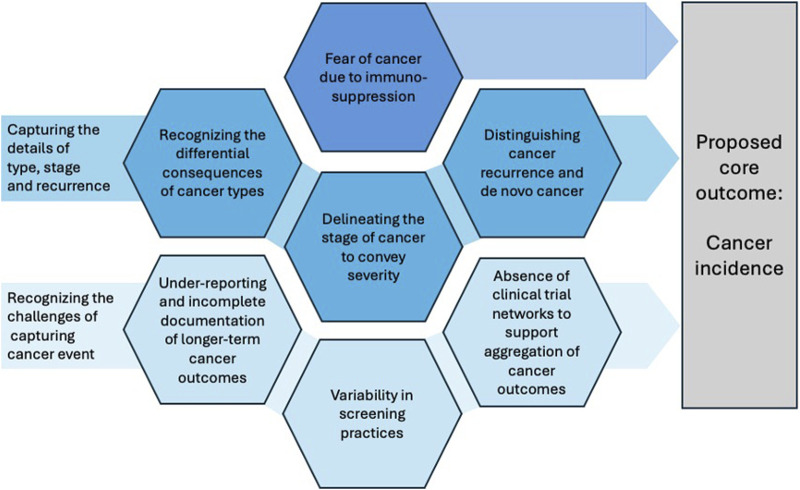
Schema of the themes derived from the consensus workshop.

We identified three themes that reflected the range of perspectives on establishing a core outcome measure for cancer: 1) fear of cancer occurrence due to immunosuppression, 2) capturing the details of type, stage and recurrence, and 3) recognizing the challenges of capturing cancer events. The themes are schematically represented in Figure 1, and described in detail below. Selected quotations for each theme are provided in Table 1. A summary of recommendations that arose from the workshop are presented in Table 2.

**TABLE 1:** Themes and illustrative quotations.

Theme	Quotations
Fear of cancer occurrence due to immunosuppression	“It’s always been something from a patient’s perspective that I’ve watched, that perhaps my kidney graft might last, but other things like cancer might get me.” P1 “I think it’s very important to the patients. Particularly, we have several here who have very long-term function, which is wonderful. And that [cancer] becomes the enemy more so than the organ function.” H1 “Now that I’m in a longer-term stage of just maintaining the transplant that I have, there’s a bit more focus on managing increased cancer risk. I Have the colorectal screening kit sitting on my desk right now. It’s really relevant for the patient perspective, because cancer is such a big scary monster in the lay population” P2 “Particularly if you’ve gone out further and further with your transplant… cancer is now the thing that you fear most rather than the kidney because the kidney is just going to carry on …if I was to get colorectal cancer now, I’d have to come off my immunosuppression, probably may lose the kidney… therefore it’s like a death sentence…it is a big worry, the cancer side of transplantation. And I think it’ll only get bigger and bigger as people’s transplants go out further and further.” P5 “The emphasis is on not losing the kidney, which is important, but I lost my father, my uncle, my brother, it’s that cancer is not addressed as aggressively as keeping the kidney and the cancer has caused the premature death of almost all of them.” P7 “Having been immunosuppressed for 9 years now, I think that the cancer occurrence should definitely be the core outcome measure, because you cannot address an illness until you know it exists, plain and simple.” P4
Capturing the details of type, stage and recurrence	Recognizing the differential consequences of cancer types
	“I actually really like the fact that we’re actually looking at all types of cancer in a kidney transplant recipient…it’s actually about trying to capture the information about all of the different types of cancers that our patient community gets affected by.” P1 “The type of cancer is extremely important…because different types of cancers have different outcomes and the risks that patients may be willing to accept will be determined on the type of cancer and the certain therapy that might be associated with.” H7 “Knowing whether this disease or if this treatment is associated with skin cancer versus brain cancer that’s a very different conversation with patients.” H7 “Definitely, the cancers are different. They have different pathologies, prognosis, different treatment patterns. I think for sure they need to be separate.” H3 “The occurrence of cancer should be the measure and [I] support more depth of information because I think from a patient carer perspective, it’s the so what? Like does it mean? There’s a tick against cancer. Well, what does that actually mean for the patient, for the carer? More information leads to improved advocacy for things like screening. And also the power is then perhaps more with the patient and the carer to initiate conversations about cancer. The nature of the cancer, the site of the cancer, whether it’s new, recurrent, I understand you’re looking for a measure, but depth of measure I think is really important as much as possible” P7
	Distinguishing cancer recurrence and *de novo* cancer
	“We’ll often be transplanting folks who’ve got a past history of cancer. That’s where the recurrence question may come in. Have they been cancer-free long enough?” H6 “I think it’s important to make a distinction between whether it is a *de novo* malignancy or whether it’s a recurrence of a malignancy that existed prior to transplant because they’re definitively separate entities.” H5 “It’s important because the *de novo* lesions are primarily driven by virally mediated cancers. And in the pre-transplant cancer population, there is a much lesser proportion of cancers that are driven by viral infection. It’s the immunosuppression that makes the difference between the two…In terms of good high quality epidemiologic data, it is far less robust dealing with preexisting malignancies than it is with a *de novo* malignancy. So, they’re markedly different types of cancers, groups of cancers that invite different questions and present significantly different epidemiologic challenges.” H5
​	Delineating the stage of cancer to convey severity
	“What does this mean for the patient’s life? What extra treatment are they going to require as a result of this cancer? Ultimately, practical things. Practical from a layperson’s perspective anyway.” P2 “We may not be able to capture all of the specifics related to a particular tumour. Still, if we are capturing the cancer category or site, that may be reasonable also to capture the cancer stage or whether cancer is metastatic or not at the time of diagnosis. I think is important considering the severity and potential treatment patients may undergo following cancer occurrence.” H5 “I think what you want to know is whether it’s metastatic. It does create difficulties for cancers like the PTLD and other haematological malignancies. But if you could just have localised or metastatic as a staging thing” H3
Recognizing the challenges of capturing cancer events	Under-reporting and incomplete documentation of longer-term cancer outcomes
	“Typically, trials are relatively short because of funding limitations and cancer may not be identified during that window of time. While I definitely support the fact that cancer, as a whole, is now being considered as an outcome measure, I would strongly encourage some element of extended follow-up for these patients, even if it’s beyond the trial period wherever possible.” H4 “I do not know that companies and other things are going to fund it for the 20 years that it would take. But if there’s a way to somehow mandate that it be linked to registries that already collect those data, that might be a way to get the answer.” H1 “I favour having this as a core outcome measure primarily because cancer is a late outcome and often not captured in other clinical trials. The longest it will go up to two to three years, so it’s really a vexing issue that does need to be tackled and is very important to the patients.” H3 “I like the idea of linking cancer databases to follow up these patients in the longer term, but for example, in low- and middle-income countries, there are no good cancer registries…And the participation of patients in clinical trials in lower and middle income countries may not be possible, [as well as] linking to a solid database to follow up the cancer.” H1 “That there’s a trial period, we have that period of time, but then for the next period of time they have the ability to pull the patient’s records. And how we go about doing that might be different in every country, as well.” P1 “Sometimes cancer is underreported. It is really not well recognised, or it is hard to keep track of. Large transplant centres may not be able to keep track properly of some of those diagnoses, whether or not they’re diagnosed years after transplant…whether or not registries would be useful just to keep track and make sure we’re not underscoring the diagnosis of cancer. It’s just hard to keep track sometimes, especially when you deal with a large population. And then we now have patients that are keeping the organs for longer time” H3
	Variability in cancer screening practices
	“It depends on the health system, whether you screen for the cancer regularly, for example, the common cancers, you do a mammogram, or you do a pap smear, or you do a colonoscopy. If you do not do it, then your incidence of cancer will be quite low. And if it’s a skin cancer, whether you send the patient to a dermatologist every year for screening” H7 “There’s an issue of the screening as well as the pace of the cancer that may not develop during that short period of time. So, I see that several challenges in reporting.” H7 “With skin and melanoma, for the most part, with good surveillance, you can manage it pretty well, speaking to someone who’s had a lot of cuts on his body. But what about those types of cancers that are not going to occur as common, and would not be picked up in testing?” P2
​	Absence of clinical trial consortia to support integration, harmonization and aggregation of cancer outcomes across studies
	“We really should bring together a group together, before we even start doing a trial to think about aggregating data at the beginning, so we can actually have information down the track. Because it is such a rare outcome with cancer, even though it is such an important outcome.” H1 “It is so difficult to power a trial to cancer…what are thoughts about bringing trialists together, researchers and patients, to a big group like this and actually design an outcome that we could actually combine all outcomes, later on down the track when the trials is finished?” H1 “Perhaps each trial will not have enough numbers to really make any difference. But then if there’s some mechanism where they can easily share data on this particular question, that may then provide what patients really care about” H2 “But I think it’s sort of like to me the two biggest goals…we want people to capture cancer and we want people to capture it uniformly so there’s the opportunity for comparison. And the easiest way to do that is to push people towards specific outcome measures that they do not have to reinvent.” H5

H, health professional, P, patient, number indicated refers to group ID from the workshop (1-7).

**TABLE 2:** Recommendations from the consensus workshop on implementing a core outcome measure for cancer in kidney transplant trials.

Overaching principle
- Ensure a balance between a simple measure that is easy to implement, and a measure that includes additional details to capture aspects important to stakeholders
For health professionals - Monitor, record and report cancer in transplant recipients to registries and trialists - Advocate for core outcomes, such as cancer occurrence, to be included in trials
For trialists/funders/pharmaceutical companies - Adopt standardised core cancer outcome measures in trials - Support the development of and invest in registries - Partner with registries to facilitate the long-term collection of cancer outcomes
For researchers - Develop clear and precise definitions of the core outcome and its components in collaboration with patients, health professionals, trialists and registry organisations - Work with journals, guideline organizations and regulatory agencies to secure endorsement and use of core cancer outcome measures - Implement core outcome sets in appropriate research settings, ensuring they are responsive to patient values - Continue to champion the inclusion of patient-centred outcomes, such as cancer occurrence (with details of cancer types, stage, and whether the malignancy is recurrent or *de novo*), in transplant research agendas
For consumers - Continue to advocate for patient-centred outcomes and reporting in clinical research and trials

## Fear of cancer occurrence due to immunosuppression

Patients feared cancer, which was described as a “big scary monster”. Some patients were not initially aware of the increased risk of cancer prior to receiving a transplant, but over time gained an understanding that their cancer risk increased with the duration, load and intensity of immunosuppression. Both patients and health professionals expressed that it was difficult navigating the balance between minimizing the risk of cancer (through reducing immunosuppression) and the fear of graft loss from rejection (through insufficient immunosuppression) – “perhaps my kidney graft might last, but other things like cancer might get me” (patient). Furthermore, there was a fear of being diagnosed with cancer as it could necessitate a reduction in immunosuppression and pose a threat to graft function, or the treatments for cancer may directly impair graft function. Both health professionals and patients were uncertain about how and when to screen for cancer due to a lack of consistent guidelines and some patients felt that they had to self-advocate to access screening for cancer.

## Capturing the details of type, stage and recurrence

### Recognizing the differential consequences of cancer types

Cancer was identified to have “different pathologies, prognosis, different treatment patterns” (health professional). Therefore, patients and health professionals believed it was informative to know about the type of cancer given that the prognosis and treatment of a cancer is specific to its type – “it’s skin cancer versus brain cancer, that’s a very different conversation with patients” (health professional).

### Delineating the stage of cancer to convey severity

Participants noted that knowing the stage of cancer was necessary to inform decisions about the treatments possible and the patient’s prognosis – “it is important considering the severity and potential treatment patients may undergo following cancer occurrence” (health professional). Patients considered the stage of cancer to reveal “practical things” – “What does this mean for the patient’s life? What extra treatment are they going to require as a result of this cancer?” (patient). However, it was acknowledged that collecting information on cancer stage could be challenging as different cancers have different staging systems. Both patients and health professionals however, supported the inclusion of staging, potentially as a binary descriptor: localized/regional versus metastatic.

### Distinguishing cancer recurrence and *de novo* cancer

Health professionals emphasized the importance of collecting information on cancer recurrence in trials to help inform clinical practice, for example, to provide data to justify and support the minimum duration a patient must be cancer free to be considered for a transplant - “we’ll often be transplanting folks who’ve got a past history of cancer. That’s where the recurrence question comes in. Have they been cancer-free long enough?” (health professional). Health professionals emphasized that first time occurring (*de novo)* cancer after transplantation could be associated with viral-mediated cancers and immunosuppression. Thus, cancer recurrence and *de novo* cancer were seen as two “groups of cancers that invite different questions and present significantly different epidemiologic challenges” (health professional) and should be distinguished when reported in trials.

## Recognizing the challenges of capturing cancer events

### Under-reporting and incomplete documentation of longer-term cancer outcomes

Participants recognized that cancer was typically a long-term outcome that could take many years to develop, be detected and may not occur during the period of a trial, which is usually conducted over a short period of time – “the trial period is limited. And the consequences [cancer] can occur in a long run” (patient). Participants supported the use of cancer occurrence as a core outcome measure, to enforce data on cancer occurrence being collected in trials - “I favor having [cancer occurrence] as a core outcomes measure primarily because cancer is a late outcome and often not captured in other clinical trials” (health professional). Patients and health professionals discussed and supported the long-term follow up of patients to capture the occurrence of cancer after trial completion through registries or data linkage – “if there’s a way to somehow mandate that [trials] be linked to registries that already collect [cancer] data, that might be a way to get an answer” (health professional). However, challenges in conducting long-term follow-up for cancer occurrence through registries were identified, including the absence of registries in some regions, particularly low- and middle-income countries, and the challenges of tracking patients between centers and over decades of follow-up.

### Variability in cancer screening practices

The lack of standardized screening protocols and access to screening were discussed as issues that may lead to variations in the reporting of cancer occurrence in different settings. Participants highlighted that there are variable screening practices based on location, local risk factors and resource availability; for example, healthcare systems with fewer resources may have reduced capacity to offer colonoscopies for colorectal cancer screening. Patients identified screening as an essential aspect of their care, because early cancer diagnosis through screening can enable timely interventions that improve patient outcomes - “with skin and melanoma, for the most part, with good surveillance, you can manage it pretty well, speaking as someone who’s had a lot of cuts on his body” (patient). However, it was also acknowledged that early cancer detection through screening may initially lead to an increased incidence, as cancers that would have been diagnosed later are being detected earlier. More importantly, the concerns of overdiagnosis were raised as screening can detect slow-growing or indolent cancers that would never have caused symptoms, morbidity or death during the person’s lifetime, causing unnecessary worry and stress.

### Absence of clinical trial networks to support integration, harmonization and aggregation of cancer outcomes across studies

Participants acknowledged that cancer events, particularly when distinguished into types, occur in small numbers in clinical trials - “[cancer] is such a rare outcome…even though it is such an important outcome” (health professional). The small number of events in individual trials makes it difficult to draw meaningful conclusions about patient outcomes without the ability to harmonize and aggregate data between trials. Combined with the long latency periods as discussed above, the small number of events led participants to advocate for the development of consortia to collate data across trials - “perhaps each trial will not have enough numbers to make any difference. But then if there’s some mechanism where they can easily share data on this particular question, that may then provide what patients really care about” (health professional). Furthermore, participants supported identifying a core outcome measure for cancer as it would further facilitate the aggregation and harmonization of data across trials - “we want people to capture cancer, and we want people to capture it uniformly so there’s the opportunity for comparison. And the easiest way to do that is to push people towards specific outcome measures that they do not have to reinvent” (health professional).

## Discussion

In this workshop, participants reached consensus that cancer occurrence should be established as the core outcome measure for cancer in kidney transplant trials. The workshop participants also acknowledged the inherent tension between minimizing immunosuppression to reduce cancer risk and maintaining sufficient immunosuppression to prevent acute rejection and graft loss. Details of cancer type, stage, and disease recurrence were identified as key factors that influence clinical outcomes and should be included. Cancer occurrence represents a significant event in a patient’s health journey and should be clearly defined and recorded. Yet, despite its relevance and importance, cancer is not uniformly and routinely captured in clinical trials and registries. There is an urgent need to harmonize and integrate long-term cancer data capture through registry-embedded trials and collaborative platforms. This will ensure consistent reporting, facilitate meaningful comparisons between trials, and support evidence-based shared decision-making to improve post-transplant outcomes.

While cancer occurrence was agreed upon as the core outcome measure, both patients and health professionals sought further details in the measure to improve its relevance and applicability. Health professionals identified that cancer encompasses a wide spectrum of pathologies, with the type of cancer and stage informing potential treatments required and prognosis. Furthermore, collecting information on whether a cancer is recurrent or *de novo* could help inform transplant practices. Patients and caregivers supported the addition of these details to enhance the utility and meaningfulness of the outcome, given the direct implications of these factors on treatment options, monitoring and prognosis. Therefore, the proposed core outcome was cancer occurrence, with specification of cancer type/site, stage as regional/localized versus metastatic and whether it is a recurrence or *de novo* cancer. It was acknowledged that an ideal core outcome measure should be simple to support its implementation and interpretation by patients and caregivers.

Although there was consensus that cancer occurrence is a critically important outcome to report in all kidney transplantation trials, a number of challenges to implementation were acknowledged that require action from clinicians, trialists and registry organizations (summarized in Table 2). Kidney transplantation trials are typically short in duration, while cancers usually take many months to years to develop and be detected. Therefore, trials in immunosuppression or other interventions that may impact cancer risk are potentially unlikely to demonstrate a difference in the effect of an intervention on cancer occurrence in short-term follow up. Furthermore, although kidney transplant recipients have a 2-3-fold increased risk of developing cancer compared to age- and sex-matched peers [1, 2], the absolute rates are initially small, with kidney transplant recipients 10, 20 and 30 years post-transplantation having cancer rates of 4.4%, 14.6% and 33.2%, respectively [12]. These challenges highlight the value of cancer occurrence as a core outcome, to allow the aggregation and harmonization of data across individual studies and populations. All stakeholders (consumers, health professionals and researchers) should continue to advocate for the inclusion of patient-centred and clinically relevant core outcomes, such as cancer incidence, in clinical trials. Attendees proposed strategies to assist the collection of data on cancer occurrence in kidney transplant recipients, including funding and expansion of registries to collect longer-term cancer outcomes and extend the follow-up of trial participants. Post-trial registry-based studies may enable clearer understanding of the risks and outcomes of cancer, compared to the findings of currently predominately retrospective studies. Challenges related to the availability and resources of registries across different settings and the reliance on health professionals for the recording and reporting of these outcomes were acknowledged. Similarly, participants felt that developing clinical trial consortia and implementing a core outcome measure for cancer occurrence would facilitate the harmonization and aggregation of results. Endorsement from journals, guideline organizations and regulatory agencies will be critical in encouraging the inclusion and reporting of core outcome measures.

In this workshop, we elicited detailed discussions regarding the development of a core outcome measure for cancer, identifying features that are critically important to patients, caregivers and health professionals. We recorded and transcribed the discussions and coded the data systematically to ensure that all ideas and experiences were captured. The preliminary findings were shared with all attendees to ensure that the reported details accurately reflected the diversity and depth of the discussions. However, this workshop has several limitations. Although we aimed to include a diverse range of stakeholders, the number of participants was relatively small, which may limit the breadth of perspectives captured. Although attendees came from 12 countries, including Indonesia, Mexico and Turkey, the majority were from Australia. However, there was substantial overlap in the themes expressed by the Australian and non-Australian participants. Furthermore, the participants were largely comprised of nephrologists, with comparatively limited representation from other disciplines such as oncology, nursing, and allied health. This may have influenced the prioritisation and framing of outcomes, particularly given the multidisciplinary nature of cancer care in transplant recipients. The workshop was conducted in English, which may have restricted participation from non-English-speaking individuals. In addition, the online format may have introduced participation bias, as patients and caregivers required access to technology, digital literacy, and sufficient resources to engage, potentially favouring individuals from higher socioeconomic settings or patients with fewer comorbidities. Consequently, the views represented may be more reflective of higher-resource contexts and may not fully capture the experiences and priorities of patients and healthcare providers in lower-resource settings.

Recommendations from the workshop represent the collective input from patients, caregivers and health professionals and will inform the development of a core outcome measure for cancer that is relevant and meaningful to all stakeholders. Establishing a meaningful and feasible outcome for cancer will support efforts to improve the frequency and consistency of cancer reporting in all kidney transplantation trials. Implementation of this core outcome measure will require support and actions from consumers, nephrologists, oncologists, hematologists and other stakeholders, including trialists, registries, guideline organizations, researchers and journals. Improved reporting of this high-priority outcome is needed to support effective shared decision-making about interventions to reduce the burden of cancer in kidney transplant recipients.

## Data Availability

The datasets presented in this article are not readily available. The de-identified coded dataset may be requested. Requests to access the datasets should be directed to Ellen Dobrijevic, ellen.dobrijevic@health.nsw.gov.au.
