# Supplementary material for: Establishing a core outcome measure for cancer in trials in kidney transplantation: a standardized outcomes in nephrology-kidney transplantation consensus workshop report
Source: Transpl Int. 2026 Jun 8;39:16181. doi: 10.3389/ti.2026.16181 (PMC13294766; doi:10.3389/ti.2026.16181)
Supplement: Supplementary file 1 [file Table1.docx]

## Supplementary materials

1. Capsule sentence summary
2. Table S1: SONG-Tx Cancer Workshop investigators
3. Table S2: Facilitator quesiton guide for breakout discussion
4. Table S3: Provided definitions of cancer outcomes

**Capsule sentence summary**

This Standardised Outcomes in Nephrology-Transplant (SONG-Tx) consensus workshop attended by 69 pateints, caregivers and health professionals from 12 countries, established that cancer occurrence would be a practical and meaningful core outcome measure for cancer in kidney transplant trials.

**Table S1: SONG-Tx Cancer Workshop investigators**

| **Health profressionals** | | | | |
| --- | --- | --- | --- | --- |
| **First Name** | **Last Name** | **Affiliation** | **Country** | **Main role(s)** |
| Abhijat | Kitchlu | University of Toronto | Canada | Physician/researcher |
| #Alejandra | Mena-Gutierrez | Wake Forest School of Medicine | United States of America (USA) | Physician/researcher |
| *Allison | Jauré | University of Sydney | Australia | Researcher |
| *Anastasia | Hughes | University of Sydney | Australia | Researcher |
| *Andrea | Viecelli | University of Zurich | Switzerland | Physician/researcher |
| Angela | Rejuso | University of Sydney | Australia | Researcher |
| *Anita | van Zwieten | University of Sydney | Australia | Researcher |
| ^Anna | Francis | Queensland Children’s Hospital | Australia | Physician/researcher |
| ^Armando | Teixeira-Pinto | University of Sydney | Australia | Researcher |
| ^Beatriz | Dominguez-Gil Gonzalez | National Transplant Organization, Madrid | Spain | Physician/researcher |
| ^Bianca | Davidson | Groote Schuur Hospital | South Africa | Physician/researcher |
| Cahyani Gita | Ambarsari | Universitas Indonesia | Indonesia | Physician/researcher |
| #Christopher | Blosser | University of Washington | USA | Physician/researcher |
| *Colm | O’Reilly | University of Sydney | Australia | Researcher |
| ^Curie | Ahn | Seoul National University | South Korea | Physician/researcher |
| *Dale | Coghlan | Flinders University | Australia | Researcher |
| David | Al-Adra | University of Wisconsin | USA | Transplant surgeon |
| David | Johnson | Univeristy of Queensland | Australia | Physician/researcher |
| Deborah | Gray-McDonald | Gold Coast University Hospital | Australia | Nurse |
| *Dharshana | Sabanayagam | University of Sydney | Australia | Physician/researcher |
| Elise | Gouin | Universite de Tours | France | Physician/researcher |
| *Ellen | Dobrijevic | University of Sydney | Australia | Physician/researcher |
| Emily | Blyth | University of Sydney | Australia | Physician/researcher |
| *Eric | Au | University of Melbourne | Australia | Physician/researcher |
| Eric | Engels | National Cancer Institute | USA | Physician/researcher |
| *#Germaine | Wong | University of Sydney | Australia | Physician/researcher |
| Greg | Knoll | University of Ottawa | Canada | Physician/researcher |
| Gregorio | Obrador | Universidad Panamericana | Mexico | Physician/researcher |
| Hatem | Amer | Mayo Clinic, Rochester | USA | Physician/researcher |
| ^Helio | Tedesco Silva | Universidade Federal De São Paulo | Brazil | Physician/researcher |
| Ibironke | Apata | Emory School of Medicine | USA | Physician/researcher |
| Jane | Tan | Standford Medicine | United | Physician/researcher |
| *Javier | Recabarren-Silva | University of Sydney | Australia | Researcher |
| Jayme | Locke | University of Alabama | USA | Transplant surgeon |
| Jeremy | Chapman | Westmead Hospital | Australia | Physician/researcher |
| ^Jolanta | Malyszko | Medical University Warsaw | Poland | Physician/researcher |
| *Jonathan | Craig | Flinders University | Australia | Physician/researcher |
| Kai Ming | Chow | Prince of Wales Hospital | Hong Kong | Physician/researcher |
| #Kenar | Jhaveri | Donald and Barbara Zucker School of Medicine Hofstra/Northwell | USA | Physicain/researcher |
| ^Kenneth | Newell | Emory University School of Medicine | USA | Transplant surgeon/researcher |
| Krista L. | Lentine | Saint Louis University | USA | Physician/researcher |
| Lai Seong | Hooi | Sultanah Aminah Hospital | Malaysia | Physician/researcher |
| Maria Irene | Bellini | Sapienza University of Rome | Italy | Surgeon/researcher |
| *Martin | Howell | University of Sydney | Australia | Researcher |
| #Naoka | Murakami | Washington University | USA | Physician/researcher |
| Nicole | Isbel | University of Queensland | Australia | Physician/researcher |
| Pablo | Fernandez-Peñas | The University of Sydney | Australia | Physician/researcher |
| Peter P. | Reese | University of Pennsylvania | USA | Physician/researcher |
| Philip | O’Connell | University of Sydney | Australia | Physician/researcher |
| Rahul | Chanchlani | McMaster Children’s Hospital | Canada | Physician/researcher |
| Rekha | Kambhampati | US Food and Drug Administration | USA | Physician/researcher |
| Ross | Francis | University of Queensland | Australia | Physician/researcher |
| Rümeyza | Kazancioglu | Bezmialem Vakif University School of Medicine | Turkey | Physician/researcher |
| Seolhyun | Lee | Stanford University | USA | Physician/researcher |
| ^Sergio | Acuna | University of Alabama at Birmingham | USA | Transplant surgeon/researcher |
| Steve | Woodle | University of Cincinnati | USA | Transplant surgeon/researcher |
| **Dual roles** | | | | |
| **First Name** | **Last Name** | **Affiliation** | **Country** | **Main role(s)** |
| Amanda | Dominello | University of Sydney | Australia | Caregiver/researcher |
| *Amanda | Sluiter | University of Sydney | Australia | Patient/researcher |
| *Chandana | Guha | University of Sydney | Australia | Caregiver/researcher |
| Luca | Torrisi | University of Sydney | Australia | Patient/researcher |
| *Nicki | Scholes-Robertson | University of Sydney | Australia | Patient/researcher |
| Shyamsundar | Muthuramalingam | South Australia Health | Australia | Patient |
| **Patients/caregivers** | | | | |
| **First Name** | **Last Name** | **Country** | | **Main role(s)** |
| Adam | Martin | Australia | | Patient |
| Adam | Akers | USA | | Patient |
| Amber | Williamson | Australia | | Patient |
| Andrew | Demaine | United Kingdom | | Patient |
| Andrew | Mansfield | Australia | | Patient |
| Bill | Wang | Hong Kong | | Patient |
| Charles | Rice | USA | | Patient |
| David | White | USA | | Patient |
| Elizabeth | Lisiecki | USA | | Patient |
| Gitthaline (candie) | Mulligan Gagne | USA | | Patient |
| Glenda | Roberts | USA | | Patient |
| John | Short | USA | | Patient |
| Kathie | Anderson | Australia | | Patient |
| Kevin | Fowler | USA | | Patient |
| Luke | Macauley | Australia | | Patient |
| Mary | Baliker | UK | | Patient |
| Tami | Sadusky | USA | | Patient |

*Facilitator or co-facilitator

#Expert working group member

^Non-attending contributor

Participants with dual roles were counted as patients/caregivers

**Table S2: Facilitator question guide for breakout discussion**

| **Definition** | Cancer occurrence = The detection of any cancer in a kidney transplant recipient during the trial period |
| --- | --- |
| **Questions** | 1. Should the occurrence of cancer be the core outcome measure?  - Why or why not? - Are there any other important aspects that should be included? E.g. new/recurrent or relapse/existing, time period for reporting  1. What would you suggest to encourage and support the implementation of this measure in trials? |

**Table S3: Provided definitions of cancer outcomes**

| **Outcome** | **Definition** |
| --- | --- |
| **Cancer occurrence** | The occurrence of any type of cancer |
| **Impact of cancer on the kidney transplant** | The cancer or cancer treatments impact the kidney transplant, for example leading to acute rejection, impaired kidney function or loss of the graft. |
| **Cancer death** | Death as a result of cancer or from cancer-related treatments. |
| **Treatment to cure cancer** | Some treatments can cure or completely remove a cancer. However, some treatments can only reduce the amount of cancer, the spread of the cancer or the symptoms form the cancer. |
| **Recurrence** | A cancer is cured or completely removed, before transplantation then later the same type of cancer is detected again after transplantation. |
| **Relapse** | A cancer is treated, and the signs or symptoms of that cancer disappear. However, some of the cells survive and later the cancer is detected again. |
| **Cancer stage** | A cancer's stage indicates how far it has spread from its original location when it is diagnosed. This can be reported as early, moderate or advanced stage or stages 1 to 4. The stage may determine which treatments are possible. |
